# Monitoring endogenous growth of open-vent volcanoes by balancing thermal and SO_2_ emissions data derived from space

**DOI:** 10.1038/s41598-019-45753-4

**Published:** 2019-06-28

**Authors:** Diego Coppola, Marco Laiolo, Francesco Massimetti, Corrado Cigolini

**Affiliations:** 1 Dipartimento di Scienze della Terra, Università degli Studi di Torino. Via V. Caluso 35, 10125 Turin, Italy; 2 Dipartimento di Scienze della Terra, Università degli Studi di Firenze. Via G. La Pira 4, 50121 Florence, Italy

**Keywords:** Volcanology, Geophysics

## Abstract

Measuring the amount of magma intruding in a volcano represents one of the main challenges of modern volcanology. While in closed-vent volcanoes this parameter is generally assessed by the inversion of deformation data, in open-vent volcanoes its measurement is more complicated and results from the balance between the magma entering and leaving the storage system. In this work we used thermal and SO_2_ flux data, derived from satellite measurements, to calculate the magma input and output rates of Mt. Etna between 2004 and 2010. We found that during the analysed period more magma was supplied than erupted, resulting into an endogenous growth rate equal to 22.9 ± 13.7 × 10^6^ m^3^ y^−1^. Notably, this unbalance was not constant in time, but showed phases of major magma accumulation and drainage acting within a compressible magma chamber. The excellent correlation with the inflation/deflation cycles measured by ground-based GPS network suggests the thermal, SO_2_ flux and deformation data, can be combined to provide a quantitative analysis of magma transport inside the shallow plumbing system of Mt Etna. Given the global coverage of satellite data and the continuous improvement of sensors in orbit, we anticipate that this approach will have sufficient detail to monitor, in real time, the endogenous growth associated to other world-wide open-vent volcanoes.

## Introduction

The rate at which deeply-sourced magma enters a magmatic system (*magma supply rate*) and the rate at which it comes out of it (*magma output rate*) are two essential parameters for estimating the endogenous growth of a volcano and for predicting its behaviour in the future^1^. These two parameters determine whether a magma chamber is in non-equilibrium steady state (i.e the magma input equal to the output), or whether it is subject to accumulation (pressurization) or emptying (depressurization) phenomena, that necessarily have a strong control on the type and timescale of surface activity^2^.

The magma supply rate (Q_in_) is classically estimated from gas flux data (mainly sulphur dioxide), by using the so-called “petrologic method”^3,4^. This method is based on a simple mass balance, from which the amount of gas emitted into the atmosphere, is proportional to the amount of magma that is supplied above the exsolution level of the volatile phase^3,4^. Hence, by measuring the SO_2_ outgassed at the vent (ϕ_SO2_, in tonnes day^−1^) and by assessing the volatile loss (i.e. ΔX_S_) suffered by the ascending magma (typically calculated as the initial volatile content in melt inclusions, minus the residual content in the groundmass), we can calculate the magma supply rate (Q_in_ in m^3^ s^−1^) as:

1 $${Q}_{in}=\frac{{\varnothing }_{so2}}{{\rho }_{m}\cdot 2{\rm{\Delta }}{X}_{S}\cdot 86.4}$$

where ρ_m_ is the dense rock equivalent (DRE) magma density (kg m^−3^) and 86.4 is the unit’s conversion factor (from tonnes day^−1^ to kg s^−1^).

The magma output rate (Q_out_) is instead calculated from repeated measurements of volumes erupted over discrete time intervals, or through a space-based thermal approach^5^. The latter, also called “thermal proxy”, derives from an energy balance of lava flows, whereby there exists a proportionality between the area, the surface temperature, and the discharge rate that feeds lava flows^6–8^. Hence, the DRE magma output rate (Q_out_), can be calculated from the satellite-derived Volcanic Radiative Power (VRP) as^8^:

2 $${Q}_{out}=\frac{{\rho }_{lava}}{{\rho }_{m}}\cdot \frac{VRP}{crad}$$

where ρ_lava_ (kg m^−3^) is the bulk densities of the lava flow and c_rad_ (J m^−3^) is an empirical best-fit parameter that relates the lava discharge rate to the thermal radiation for any given rheological case^8^.

To these two rates of magma transport, a third parameter of vital importance needs to be considered to fully constrain the magma budget of a volcano. This is represented by the rate of magma accumulation, or withdrawal (dQ_m_ = Q_in_ − Q_out_), that determines if a volcano is inflating or deflating^9,10^. However, because magma is compressible, only a fraction of the net magma volume entering or leaving the chamber (ΔV_m_ = V_in_ − V_out_) will be accommodated by the expansion/contraction of the chamber itself (ΔV_c_), so that^10,11^:

3 $${\rm{\Delta }}{V}_{m}=(1+\frac{{\beta }_{m}}{{\beta }_{c}})\cdot {\rm{\Delta }}{V}_{c}$$

where *β*_*m*_ and *β*_*c*_ are the compressibilities of magma and chamber, respectively. In turn, subsurface volume changes (ΔV_c_) are generally constrained from the surface displacements observed by geodetic techniques, such as GPS and InSAR^12^, although source geometry and material properties can also play a fundamental role in modulating the ground deformation^11,13^.

It is therefore clear that there must be a direct correlation between the rates of degassing, eruption and deformation, through which the magma budget is satisfied (eq. 3).

At *closed-vent volcanoes* (i.e. volcanoes with sealed conduits that may exhibit edifice-wide long-term ground uplift due to reservoir pressurization^14^), the classic model of the “volcano deformation cycle”, is generally operative^2^. According to this model, during inter-eruptive periods, the magma output rate (Q_out_) is equal to zero, so that all the magma supply (Q_in_) gradually inflates a chamber beneath the volcanic edifice, causing uplift. Once the eruption starts (i.e. once a critical pressure is reached at which point the chamber ruptures), Q_out_ becomes much higher than Q_in_, so the volcano deflates, because of withdrawal from the magma chamber^9,10^. In some cases, this long term inflation/deflation cycle has shown predictable behaviours, allowing successful forecast of eruptions^15–17^.

On the other hand, it is now well established that *open-vent volcanoes* (i.e. volcanoes characterized by persistent degassing and continual emission of magmatic-related products directly to the atmosphere^18^), emit an excess of gas to that contained in the erupted magma^19,20^. This unbalance, commonly known as “excess degassing”^19,20^, has been ascribed to phenomena of endogenous or cryptic growth, in which some unerupted magma is continuously intruded in the volcano edifice, or ascends, degasses and then sinks back down the conduit to mix with a deep reservoir^1,4,21–25^. In most *open-vent volcanoes* this large unbalance does not translate into significant long-term surface deformation, but only into short-term, localized deformation related to shallow conduit processes^14^. For example, short-term deformation cycles have been observed in association with Strombolian or Vulcanian eruptions^26,27^ as well as with variations in the lava lake level^28^. Also, is not uncommon to observe deflation phenomena during major eruption phases, that temporary drain the magma chambers^9,29^ or the upper portion of the plumbing system^30–32^. According to some authors the lack of long-term inflation at these *open-vent volcanoes* cannot be explained by magma reservoirs being too deep to create detectable uplift, but rather by the possibility of magma to rise toward the surface without pressurizing the magma reservoirs^14^.

Therefore, is still unclear how the large amount of unerupted magma ΔV_m,_ characterizing open-vent volcanoes is related to the subsurface volume changes ΔV_c_ inferred from ground deformation, although the two volumes are clearly linked (eq. 3).

Mt. Etna has been object of several works focused on the balance between supplied and erupted magma, all showing that only a small fraction of magma entering the degassing zone (3–4 km b.s.l.), is actually extruded^23,29,33^. Most studies have examined this *open-vent* behaviour over periods of decades, suggesting an overall endogenous growth by intrusion and the creation of a cryptic plutonic complex within Etna’s sedimentary basement^23,29,33,34^. Some detailed magma budget was also presented for three individual eruptions of Mt. Etna^35^, suggesting that over timescale of weeks, the balance between input and output rates is variable and much more complex than previously thought. However, this study did not consider the magma budget during the periods between eruptions, thus lacking one half of the whole history. First clues on Etna’s magma budget during a sequence of short-lived paroxysms^36^ suggests that the majority of the magma erupted during these events was likely stored within the conduit, having gone through extensive degassing for days to weeks prior to the paroxysms. Notably, all these works^23,29,33–36^ focused on the magma budget of Mt. Etna over different timescale (from decades to days), but none looked at the relationships with the volcano deformation over the same timescales.

On the other hand, ground and satellite measurements of deformation suggest that Mt. Etna is affected by an overall long-term inflation trend, periodically interrupted by more or less important deflation periods^29,37–40^, a pattern somehow similar to the classic *closed-vent* volcano deformation cycle. One work first correlated this deformation pattern with the long-term bulk magma budget characterizing the 1993–2005 period^29^, but it does not have the sufficient temporal detail to explore the validity of the above model in the mid-, or short-term.

**Figure 1:**
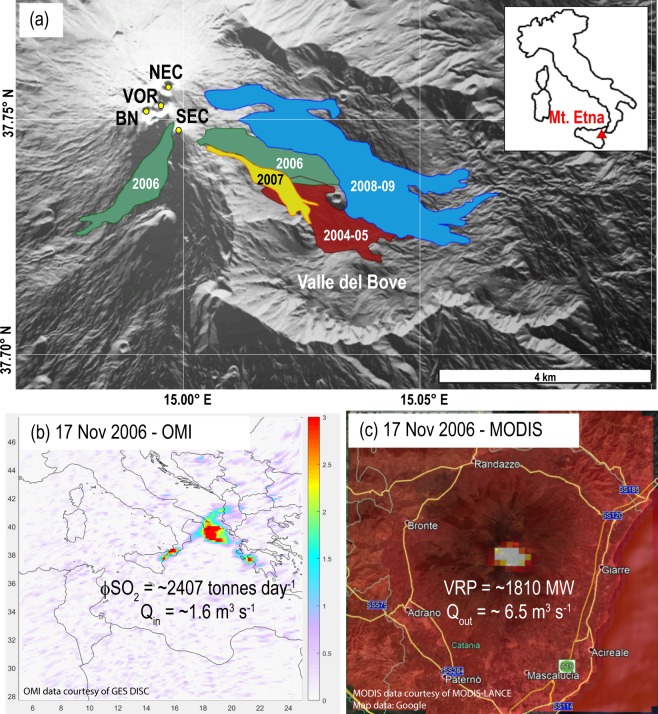
(**a**) Simplified map of Etna summit craters and main lava flows emplaced between 2004 and 2010. (shaded relief map derived from Tinitaly Digital Elevation Model^70^; http://tinitaly.pi.ingv.it/). (**b**) Example of OMI image acquired over Mt. Etna on 17 November 2006 showing an SO_2_-rich plume (colorscale refers to Dobson Units; DU) drifting hundreds of kilometres toward north-east. Based on eq. 1 the sulphur flux rate measured at that time is consistent with a magma supply rate Q_in_, equal to 1.6 ± 0.5 m^3^ s^−1^. (**c**) Coeval MODIS-MIROVA image showing the thermal anomaly produced by the lava flow descending on the Valle del Bove. According to eq. 2, the thermal flux recorded on 17 November 2006 was produced by a magma output rate, Q_out_, equal to 6.5 ± 1.9 m^3^ s^−1^. The evident unbalance between Q_in_ and Q_out_ suggests the empting of a shallow magma chamber consistent with a stage of deflation of the volcano edifice, as measured by GPS network (cfr. Fig. 4). (OMSO2 Level 2G data courtesy of Goddard Earth Sciences Data and Information Services Center - GES DISC; https://disc.gsfc.nasa.gov/). (MODIS Level 1b radiance data courtesy of LANCE-MODIS system https://lance-modis.eosdis.nasa.gov/; Base Map data courtesy of Google).

In this work we used satellite thermal data, derived from Moderate Resolution Imaging Spectroradiometer – MODIS (Fig. 1a) and SO_2_ flux data, derived from Ozone Monitoring Instrument – OMI (Fig. 1b), to constrain the magma supply and output rates (methods) characterizing the activity of Mt. Etna between 2004 and 2010 (Fig. 1c). These two independent datasets provide for the first time a clear, quantitative view of the magma accumulation and withdrawal processes affecting the volcano plumbing system at different timescales. These results are finally compared with the deformation data measured by the CGPS network during the same period^38,39^ and highlight the great potential of using combined satellite data to monitor quantitatively the endogenous growth of open-vent volcanoes.

## Activity of Mt. Etna between 2004 and 2010

After ∼20 months of quiescence, and without precursory seismicity or ground deformation, a new eruption started on 7 September 2004, through a complex set of fractures opened in the summit area of Mt. Etna^39^. The eruption produced a voluminous lava flow (40–64 × 10^6^ m^3^) within the Valle del Bove (Fig. 1c) and maintained low rate, steady conditions up to the end, occurring on 8^th^ March 2005^41,42^. Geophysical, geochemical and petrologic data suggest that the magma feeding the 2004–2005 activity was likely to have been stored in the shallow plumbing system during the 2000 and 2001 activity, where it experienced volatile loss and extensive crystallization^41,43,44^.

Few months after the 2004 eruption, between November 2005 and January 2006, the amplitude of volcanic tremor increased abruptly, although neither effusive nor paroxysmal activity was observed at the summit vents^45^. This event was associated with mild inflation of the summit of the volcano and with increased SO_2_ flux, allowing some authors to interpret it as a failed eruption, resulting from recharging of the volcanic feeder at depth (>3 km b.s.l.)^45^. Following this intrusive event, minor explosive activity occurred in the summit area (Bocca Nuova – BN- and Voragine –VOR - craters; Fig. 1c) during 2006, whereas the Northeast Crater (NEC; Fig. 1c) was the site of deep-seated explosions, which increased in early July 2006. The heightening of NEC activity was the prelude to the 2006 eruption that started on 14 July and that was characterised by two main phases of activity^46^. The first phase (14–24 July) lasted 10 days during which Strombolian and effusive activity (emitting 3–6 × 10^6^ m^3^ of lavas) took place along a short fissure on the lower ESE flank of the SEC cone^47^. The second phase lasted from 31 August until 14 December and consisted of 20 eruptive episodes at or near the summit of the SEC cone producing ash plumes. From 12 October onward, an almost persistent effusive activity took place from a number of vents opened on the south of SEC^46^. (Fig. 1c). As a whole the 2006 eruptive activity produced two main lava fields emplaced on the south-west and on the south-east flank of the volcano, with a total lava volume of 20–37 × 10^6^ m^3^ ^42,46^.

After the end of the 2006 eruption, a recharge phase was indicated by continuous inflation showed by GPS measurements^38,39^. The recharge phase was accompanied by seven short-lived lava-fountaining episodes that erupted a total of ∼12 × 10^6^ m^3^ of lava, and marked the shifting of activity from the SEC to the newly formed new SE crater (NSEC)^48–50^. The last powerful episode occurred on 10 May 2008, only 3 days before the onset of the 2008 eruption^51^. Unlike the previous two eruptions, the initial phase of the May 2008 eruption was preceded and accompanied by strong seismic release and by marked deformation of the volcano flanks^52^.

Moreover, in this case, the eruptive fissure propagated inside the Valle del Bove, producing the long-lived 2008–2009 eruption that lasted 419 days (the longest eruption since 1991–1993) and that emitted 68–74 × 10^6^ m^3^ of lava^42,53^ (Fig. 1c). After the end of this eruption Mt. Etna was characterized only by continuous degassing and by sporadic minor explosive phenomena for 17 months^49^. Overall, the 2004–2010 activity of Mt. Etna was composed of 3 major eruptions, 1 intrusive event and 7 episodes of lava fountaining, that altogether erupted 151–173 × 10^6^ m^3^ of lava^42^.

## Results

**Figure 2:**
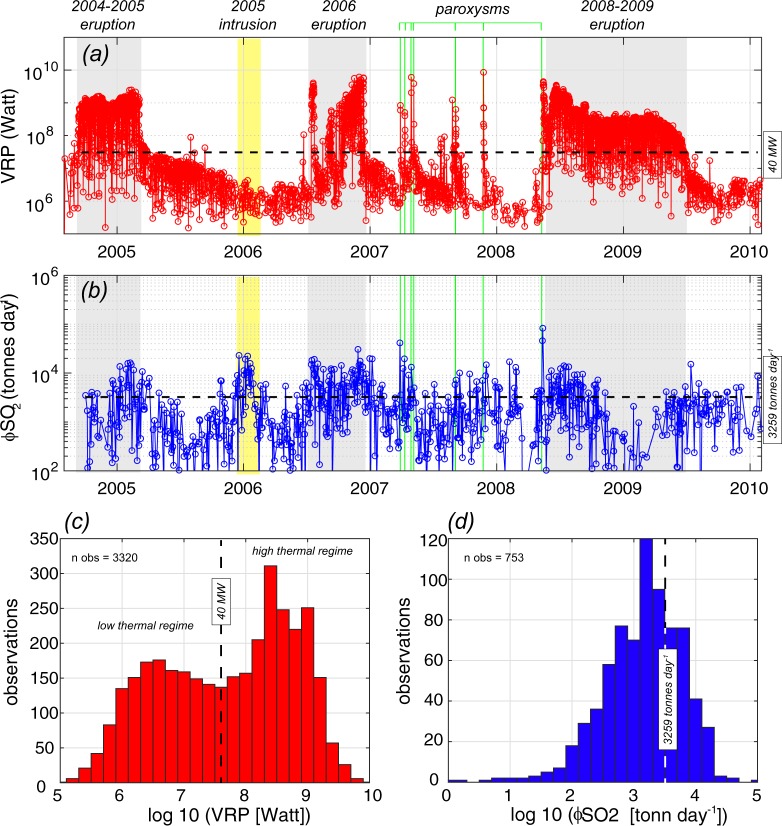
Time series of (**a**) Volcanic Radiative Power - VRP and (**b**) sulphur dioxide flux - ϕSO_2_ retrieved over Mt. Etna between September 2004 and February 2010 (from MODIS and OMI data, respectively; see methodology). Grey fields outline the occurrence of the three major eruptions accompanied by lava effusion. Yellow field outlines the failed eruption in winter 2005–2006^45^. Green bars outline the seven fountaining episodes (paroxysms) occurred between 2007 and 2008^48^. (**c**) Frequency of distribution for VRP datasets (log-transformed data). Note the bimodal distribution of log VRP marking the transition from the low to the high thermal regime for VRP = 40 MW (balck dashed line in (**a**)). (**d**) Frequency of distribution for ϕSO_2_ dataset (log-transformed data) showing unimodal pattern and arithmetic mean at log SO_2_ = 3.5 (ϕSO_2_ = 3259 tonnes day^−1^).

The time series of Volcanic Radiative Power (VRP) recorded between 2004 and 2010 (methods) outlines a continuous thermal emissions (∼1.65 thermal detections per day) with VRP ranging from less than 1 MW to ∼8.5 GW (Fig. 2a). The 3 major eruptions (2004–2005, 2006, and 2008–2009; grey fields in Fig. 2a) as well as the 7 paroxysmal episodes occurred on 2007 and 2008 (green dashed lines in Fig. 2a) are clearly visible and they separate from the low and continuous thermal emission that characterise the entire period investigated. The statistical analysis of the VRP (log-transformed), indicates a bimodal distribution, with two distinct regimes within the entire population of data (n_obs_ = 3320; Fig. 2c). The low thermal regime, characterized by VRP < 40 MW ( < 7.6 log-transformed), was typical of inter-eruptive periods, when the thermal source was likely produced by weak strombolian activity, hot degassing cracks within the summit craters, as well as by the cooling of previously emplaced lava flows (during the post-eruptive phases; Fig. 2a). Conversely, the high thermal regime (VRP > 40 MW) corresponds to periods of major effusive activity, which also includes the 7 lava-fountaining episodes occurred in 2007 and 2008 (Fig. 2a). It is thus clear that a change from low to high thermal regimes (peaking at 4 MW and 252 MW, respectively) corresponds to a transition from open-vent to effusive activity, in manner similar to that already observed at Stromboli volcano^54^.

The SO_2_ flux data (Fig. 2b) also indicate a continuous degassing between 2004 and 2010 (∼0.37 SO_2_ detections per day), but with an unimodal distribution of the data (n_obs_ = 753; Fig. 2d). The average *ϕSO*_2_ obtained from the OMI data (Methods) is equal to 3259 tonnes day^−1^, in excellent agreement with the flux measured by ground measurements during 2005–2008^55^ (see methods). It is worth nothing that during the inter-eruptive phases, the average flux was 3023 tonnes day^−1^, while during the 3 effusive phases (grey fields in Fig. 2) the average *ϕSO*_2_ raised to 4301 tonnes day^−1^. This suggests that the effusive eruptions of Mt. Etna were characterized by a bulk increase of the degassing rate of only ∼42% with respect to the persistent, long term emission.

Notably, the failed eruption that occurred during the winter of 2005–2006^45^ (yellow field in Fig. 2) was accompanied by a monthly-long pulse of *ϕSO*_2_, while there were no anomalous thermal emissions recorded in the VRP time series (the cooling trend remained essentially unperturbed; Fig. 2a). In contrast, all the lava-fountain episodes are evident in the VRP time series (Fig. 2a), but not particularly in the *ϕSO*_2_ time series (with exception of those occurred on 29 March 2007 and 10 May 2008).

This may be due to the impulsive nature of the phenomenon (i.e. duration less than 1 day, on average) which coupled with the low sampling rate of OMI (1 image per day) makes challenging the detection of all short-lived SO_2_ emissions associated to these episodes^36^.

In general, it can be observed that both thermal and gas emissions have been continuous during 2004–2010 (Fig. 2a,b), remarking the persistent open-vent behaviour of Mt. Etna. Nevertheless, the thermal flux shows wide and evident increases during major eruptions (almost two orders of magnitude), leading to a bimodal distribution of VRP (Fig. 2c). On the contrary, *ϕSO*_2_ shows an unimodal distribution (Fig. 2d) and a more homogeneous trend, with smoothed, monthly-long oscillations accompanying the major eruptions and the intrusive episode (Fig. 2b).

## Discussion

The magma supply rate and the magma output rate have been estimated using eqs 1 and 2, whose detailed parametrization is described in the method section. However, some aspects must be clarified regarding the interpretation of these parameters and their volcanological significance at open-vent volcanoes.

The magma supply rate calculated by eq. 1 (Q_in_) is in fact a “magma degassing rate”, since it provides an estimate of the quantity of magma that rises above the exsolution level of SO_2_ and degasses^1,19,23^. Therefore, it should be interpreted in a broad sense, as the rate of magma entering the shallow portion of the magma system where a convective degassing cell is operative (about 3–4 km b.s.l. for Etna^4,23,33^).

On the contrary, the magma output rate (Q_out_) calculated through eq. 2 provides an estimate of the lava discharge rate during the effusive phases^8^. However, during phases of open-vent activity, the magma output rate assumes an ambiguous meaning, and should be considered an “apparent output rate”, since there is no net extrusion of lava. In this case, the application of eq. 2 can still be used to infer the rate at which the magma reaches the surficial levels in the conduit (i.e., few tens/hundreds meters at most), where it radiates and cools before being cycled back^8,36,56^. In this scenario, the threshold separating the low thermal regime (open-vent activity) from the high radiating regime (effusive activity) indicates that an ascending volumetric flux of 0.16 ± 0.05 m^3^ s^−1^ represents a physical barrier for the convective magma transport within the conduit(s) of Mt. Etna. Above this threshold the magma can no longer be recycled in the conduit and must be partially or completely extruded.

Accordingly, the two magma fluxes (Q_in_ and Q_out_) sample the rate of magma transport at different depths^56^, being the SO_2_‐derived flux relative to the magma circulation above the exsolution level of SO_2_, and the IR‐derived flux relative to very shallow (i.e. during open-vent) or surficial (during effusive activity) levels.

**Figure 3:**
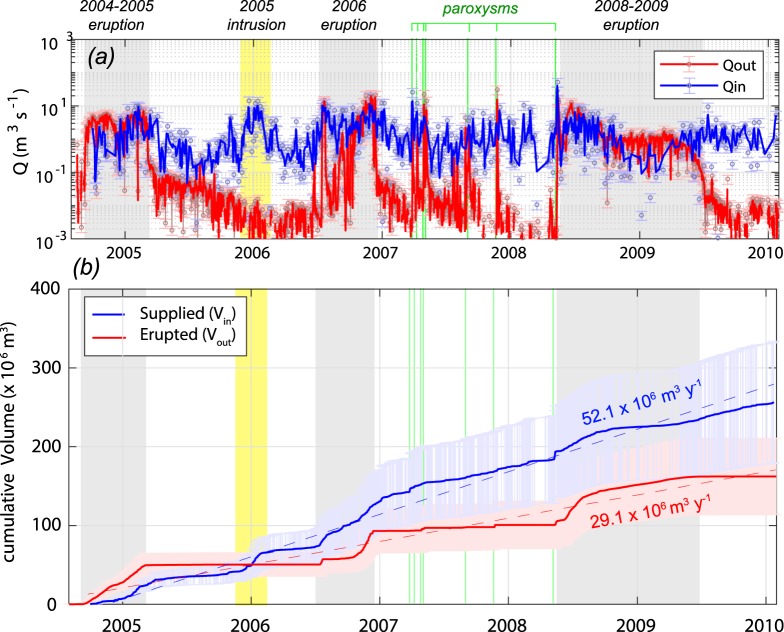
(**a**) Time series of magma input (Q_in_) and output (Q_out_) rates, derived from SO_2_ and thermal data (eqs 1 and 2; see methods for details). (**b**) Cumulative volumes of magma supplied (V_in_) and erupted (V_out_) throughout the 2004–2010 period. Uncertainties of ±30% in fluxes/volumes values (see methods) is shown by the respective errorbars. Coloured fields and bars as in Fig. 2.

The time series of Q_in_ and Q_out_, as well as the respective cumulative volumes (V_in_ and V_out_) estimated for the 2004–2010 period, are shown in Fig. 3. The direct comparison of these rates clearly shows the overall unbalance due to the excessive degassing of Mt. Etna, with supply rate generally exceeding the output rate. This unbalance is particularly evident during inter-eruptive periods, while it is cancelled, or sometimes reversed, during the major eruptive phases (Fig. 3a).

**Figure 4:**
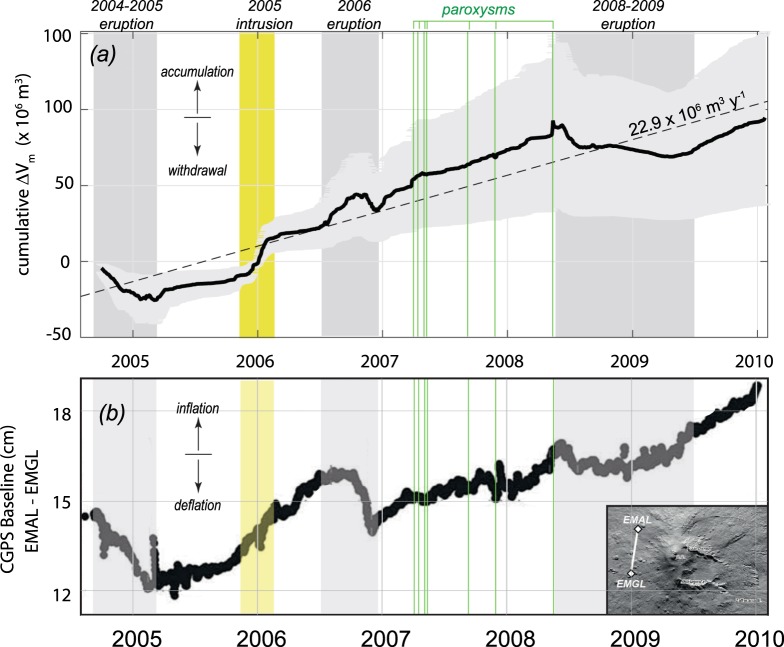
(**a**) Time series of cumulative ΔV_m_, retrieved from magma budget calculations (ΔV_m_ = V_in _− V_out_); (**b**) time series of the length variations between the EMAL and EMGL CGPS stations (inset), modified from Bruno *et al*.^38^. Note the good correlation between the overall trends of the two datasets, as well as the excellent correspondence between the main phases of magma accumulation/withdrawal (**a**), leading to coeval and coherent inflation/deflation cycles (**b**). Coloured fields and bars as in Fig. 2.

In general, our volume calculations (Fig. 3b) indicate that between October 2004 and February 2010, 257.0 ± 77.1 × 10^6^ m^3^ of magma entered the system with an average Q_in_ of 1.65 ± 0.49 m^3^ s^−1^ (52.1 ± 5.6 × 10^6^ m^3^ y^−1^), while 164.0 ± 49.2 × 10^6^ m^3^ came out of the system with an average Q_out_ of 0.92 ± 0.28 m^3^ s^−1^ (29.1 ± 8.73 × 10^6^ m^3^ y^−1^). This unbalance results in a net magma accumulation rate equal to 22.9 ± 13.7 × 10^6^ m^3^ y^−1^ (Fig. 4a), a value similar to that measured during 1975–1995 period (∼25.1 × 10^6^ m^3^ y^−1^)^23^, but lower than the accumulation rate characterizing the 1993–2005 period (∼64.1 × 10^6^ m^3^ y^−1^)^33^.

Although the long-term magma budget indicates a general accumulation of magma, the trend shows mid- and short-term variations of ΔV_m_ that can be compared with the deformation data reported for the same period. In this regard, we have used the published baseline distance between two CGPS stations (located on the west side of Etna; inset in Fig. 4b), which highlight the deformation pattern characterizing Mt. Etna activity between 2004–2010^38^ (Fig. 4b).

This comparison shows an excellent correlation between the two datasets, suggesting that the magma budget retrieved from SO_2_ and thermal data is closely linked to the volcano deformation. All the main stages of inflation (positive ΔV_m_) are actually corresponding to periods of “excess degassing” produced by magma accumulation at depth, without net extrusion of magma. These stages essentially correspond to the open-vent activity, or to periods characterized by magmatic intrusion, like the one occurred in the winter 2005–2006 (yellow field in Fig. 4). On the other hand, all periods of magma drainage (negative ΔV_m_) occurred during deflation stages, and in particular during specific periods of the main effusive eruptions (grey fields in Fig. 4a).

**Figure 5:**
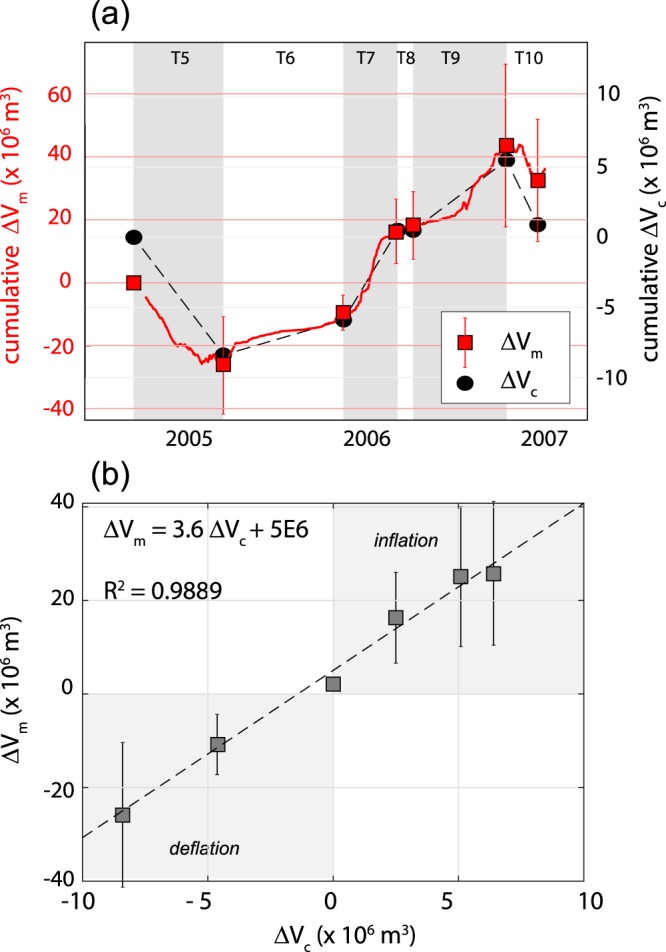
(**a**) Time series of cumulative ΔV_m_, retrieved from Q_in_ and Q_out_ (red line), and cumulative ΔV_c_, inferred from deformation data^39^ (black dashed line) for the 2004–2007 period. Six stages of inflation/deflation are identified and named (from T5 to T10) after Palano *et al*.^39^. (**b**) Correlation between ΔV_m_ and ΔV_c_ for the six analysed stages. The ratio ΔV_m_/ΔV_c_ = 3.6 is consistent with a magma chamber filled by bubble poor basaltic magma^10,11^.

By using similar GPS analysis, other authors^39^ identified 6 stages of inflation/deflation occurred between 2004 and 2007 (named from T5 to T10; Table 1). For each stage they estimated the depth (D) of the deformation source as well as the magma chamber volume changes (ΔV_c_) that better explain the ground deformation pattern (Fig. 5a). These data indicate the presence of a magma chamber at 3.5–5.5 km b.s.l. where the pressurization/depressurization occurs. The volumes changes (ΔV_c_) inferred for each stage span from −8.4 × 10^6^ m^3^ (T5) to +6.4 × 10^6^ m^3^ (T7), and compare very well with the volumes calculated from the magma budget approach (ΔV_m_; Table 1). This excellent correlation (R^2^ = 0.989; Fig. 5b) indicates a ΔV_m_/ΔV_c_ ratio spanning from 2.4 to 6.5, with a best-fit value equal to 3.6 (Fig. 5b). As described previously this ratio describes the compressibility of the system (eq. 3), which for bubble poor basaltic magmas stored at shallow crustal levels spans from 1.2 to 7.7^10,11^. These results are thus consistent with the occurrence of efficient magma convection in the upper magmatic systems of Mt. Etna allowing degassed magma to sinks back and accumulate within the deforming magma chamber.

**Table 1:** Synthesis of magma budget volumes (ΔV_m_) and subsurface volume changes (ΔV_c_) for the six inflation/deflation stages occurred at Mt. Etna between 2004 and 2007

Phase*#*	activity	trend	beginning#	end*#*	Depth^a^	ΔV_c_^(a)^	cum ΔV_c_	ΔV_m_ = V_in_ − V_out_^b^	cum ΔV_m_	Δ*V*_*m*_*/*Δ*V*_*c*_*#*
					*m b.s.l*.	*m*^*3*^ × *10*^*6*^	*m*^*3*^ × *10*^*6*^	*m*^*3*^ × *10*^*6*^	*m*^*3*^ × *10*^*6*^	
T5	eruption	*deflation*	07/09/2004	10/03/2005	5450	−8.4	−8.4	−25.9 (±15.6)	−25.9 (±15.6)	3.1 (±1.9)
T6	open-vent	*inflation*	10/03/2005	12/11/2005	4804	2.5	−5.9	16.2 (±9.8)	−9.6 (±5.8)	6.5 (±3.9)
T7	intrusion	*inflation*	12/11/2005	03/03/2006	4503	6.4	0.5	25.7 (±15.4)	16.1 (±9.7)	4.0 (±2.4)
T8	open-vent	*inflation*	03/03/2006	04/04/2006	N.D	N.D.	0.5	2.1 (±1.3)	18.2 (±10.9)	N.D.
T9	open-vent	*inflation*	04/04/2006	13/10/2006	5064	5.0	5.5	25.1 (±15.1)	43.3 (±26.0)	5.0 (±3.0)
T10	eruption	*deflation*	13/10/2006	16/12/2006	3662	−4.6	0.9	−10.8 (±6.5)	32.5 (±19.5)	2.3 (±1.4)

^a^From Palano *et al*.^39^.

^b^This work.

These results have a series of important implications for the long- and short- term monitoring of volcanoes showing persistent activity.

Firstly, we demonstrated that the endogenous growth of Mt. Etna volcano can be quantified in great detail by means of satellite-derived thermal and SO_2_ flux data, and that the resulting magma budget (ΔV_m_) is compatible with the volume changes (ΔV_c_) inferred by deformation measurements, and with the bulk compressibility of the magmatic system.

Starting from this evidence the following considerations can be drawn for any open-vent volcano:

- each open-vent volcano that degasses more magma than it emits, grows in an endogenous way;
- this growth can be monitored by means of satellite-derived thermal and SO_2_ measurements (allowing the calculation of ΔV_m_; eqs 1 and 2), or by means of deformation measurements (allowing the calculation of ΔV_c_; eq. 3);
- The ratio between ΔV_m_ and ΔV_c_ is a function of the compressibility of the system and can be used to to infer depth of the accumulation zone as well as the magma and the crustal parameters;
- The continuous monitoring of ΔV_m_ and ΔV_c_ allows to quantify the shallow magma budget of the system. Each temporal decorrelation between these two volumes is indicative of a change in (i) position of the magma accumulation zone (ii) initial volatile’s content (iii) thermo-elastic properties of the system.
- if open-vent activity is not accompanied by long-term detectable deformation (i.e. Merapi, Popocatépetl, Colima^14^) the endogenous growth possibly occurs within a very compliant crust or at shallow depths, or in correspondence of a bubble-rich, highly compressible magma chamber, characterized by a high ΔV_m_/ΔV_c_ ratio^13^.

We also demonstrated that thermal and gas emissions are not necessarily correlated throughout effusive activity^3^, nor during open-vent activity (Fig. 2). This implies that, even at persistently degassing volcanoes, the high-temperature thermal anomalies measured by satellite^57^ (i.e. Te > 600 K), are not necessarily correlated to the emission of gas itself, as hypothesized by^58^. In fact, the magmatic intrusions occurred between December 2005 and January 2006, although it produced an evident pulsation of SO_2_ flux from the summit craters (>20 ktonnes day^−1^), it was not accompanied by evident thermal anomalies, just because the magma remained at depth (>3 km b.s.l.^45^). Accordingly, we here stress that also at persistently degassing volcanoes, the detection of MIROVA thermal anomalies, advise that the magma column has reached the surface, or is at very shallow depth.

A first recent comparison of thermal anomalies, degassing, and surface deformation, derived from satellite data at 47 of the most active volcanoes in Latin America, provides clear evidence of the potential of applying this approach at regional scale^59^. Equations 1, 2 and 3 can be applied in near real time if appropriate parametrization has been validated such as for Mt. Etna. We believe that in the future, the technological advancement represented by the new infrared, ultraviolet and microwave satellite’s sensors will allow a sufficient degree of detail for monitoring on a daily basis the endogenous growth of open-vent volcanoes and, more generally, the efficiency of magma transport within the magmatic plumbing systems.

## Methods

### Volcanic Radiative Power (VRP) via MODIS-MIROVA data

Thermal emissions of Mt. Etna, have been obtained by analysing the data provided by the Moderate Resolution imaging Spectroradiometer (MODIS). Two MODIS sensors, in orbit since February 2000 and May 2002 (on board of Terra and Aqua NASA’s satellites, respectively) provide, on average, 4 images per day of the entire globe, with a spatial resolution of 1 km in the infrared. The data were processed through the MIROVA system^57^, an automatic volcanic hotspot detection system, based on the analysis of Middle Infrared (MIR) images. This system allows to detect, locate and quantify the thermal emissions sourced by volcanic activity by calculating the Volcanic Radiative Power (VRP). The VRP is a measurement of the heat flux radiated almost exclusively by hot lava surfaces (i.e. having effective temperature above 600 K) and is calculated via the MIR method^60^ as:

4 $$VRP=18.9\cdot {A}_{pixel}\cdot \sum _{i=1}^{npix}{({R}_{\mathrm{MIR},alert}-{R}_{{\rm{M}}IR,bk})}_{i}$$

where R_MIR,alert_ is the pixel integrated MIR radiance of the i^th^ alerted pixel, R_MIR,bk_ is the MIR radiance of the background, A_pixel_ is the pixel size (1 km^2^ for the resampled MODIS pixels), and 18.9 is a constant of proportionality^60^. The VRP has an uncertainty of ±30%^60^ which cover the possible insulation conditions (integrated surface temperature from 600 K to 1500 K) of the observed lava flow (not shown in Fig. 2a for graphical clarity).

In this work, we analysed 8903 images acquired over Mt. Etna between 1 August 2004 and 31 January 2010. Within this dataset MIROVA detected a thermal anomaly in 3320 images (∼37%) allowing the construction of the VRP time series shown in Fig. 2a. The presence of clouds (or ash plumes), as well as the poor viewing geometry conditions may prevent or affect the thermal signal detected by the satellite^57^. In volcanoes that exhibit persistent thermal anomalies (i.e more than one a day; as for Etna) a practical way to overcome this attenuation is to considered the maximum daily VRP (Supplementary Dataset) as the most reliable value to calculate the daily magma output rate^57^. The latter has been calculated by using the eq. (2), where a single coefficient, called radiant density (c_rad_ in in J m^−3^) describes the relationship between volumetric and radiant flux appropriate for the observed eruption^8^. An exhaustive review of the application of this method over basaltic lava flows is provided by^61^.

Here we used a radiant density of 2.5 × 10^8^ J m^−3^ which has been already calibrated for Etnean lava and provides estimates of magma output rate with an uncertainty of ±30%^8,57^. According to^57^, this uncertainty embeds all the possible emplacement conditions (rheological, topographic and insulation) that characterize the Etnean lava flows. In order to obtain dense rock equivalent magma output rates (Q_out_), we assumed ρ_m_ = 2600 kg m^−3^, and ρ_lava_ = 2350 kg m^−3^ to give a bulk flow vescicularity of about 10%^62^.

### SO_2_ flux (*ϕ*SO_2_) via Ozone Monitoring Instrument (OMI)

The OMI is one of the four instruments on board of AURA platform dedicated to monitor solar backscatter radiation at wavelengths spanning from 270 to 500 nm (visible and ultraviolet). It provides a daily global coverage in 14 orbits since the 1^st^ October 2004. Each image has a complete swath of 2600 km and a nominal pixel spatial resolution of 13 × 24 km at nadir. Here we used the OMSO2 Level 2G (0.125° × 0.125°) global product^63^ that contains estimates of the total SO_2_ vertical column density (VCD_SO2_; in D.U.), assuming different centres of mass altitudes (CMAs), at 0.9 km (Planetary Boundary Layer, PBL), 3 km (Lower tropospheric, TRL), 8 km (Middle tropospheric, TRM), and 17 km (Lower stratospheric, STL), respectively. Since 2010, a technical issue on the instrument’s field of view makes certain rows of the OMI swath unusable for SO_2_ retrieval. This led the occurrence of 2–3 days-long gaps between consecutive OMI observations that corrupt the quantification of the long-term degassing budget. Here, we processed OMI data acquired between 1^st^ October 2004 to 1^st^ February 2010, consisting of 1831 images.

To detect SO_2_ emitted from Mt. Etna we adopted the same procedure described in^64^, composed by the following steps:

(i) Extraction and cropping of mask of 20° × 20° centred on Mt. Etna, for any CMA product;
(ii) Calculation of a contextual threshold at PBL level, defined by T_so2_ = μ + 3σ, where μ and σ represent the mean and the standard deviation of those pixels within the mask having VCD_SO2_ < 1 D.U (at PBL level);
(iii) Recognition and definition of groups of adjacent pixels (clusters) that positively exceed this threshold;
(iv) Visual inspection and manual selection of all the clusters attributed to the Etna’s plume.

For any image where at least one cluster was selected, we calculated the SO_2_ mass burden (M_SO2_, in tonnes), at PBL, TRL, TRM and STL layers, by using the following equation, as proposed by^65^:

5 $${M}_{SO2}=0.0285\cdot {A}_{pix}\sum _{i=1}^{npix}VC{D}_{SO2}$$

where *npix*, *A*_*pix*_ and VCD_SO2_ represent, the number of alerted pixels, the pixel area (in km^2^) and the vertical column density of SO_2_ (in D.U.), of the selected plume.

For Mt. Etna plume we used the SO_2_ mass retrieved for PBL and TRL layers, because the information on the typical altitude reached by the volcanic plume related to the activity of the analysed volcanoes (less than 5 km). In particular, we used the PBL level for the most of the cases which provide good results to quantify passive degassing emitted by open vent activity^66^. However, during more powerful plume emissions, hereby defined as plumes producing more than 20 ktonnes in the PBL, we used the mass burden calculated for TRL layer.

**Figure 6:**
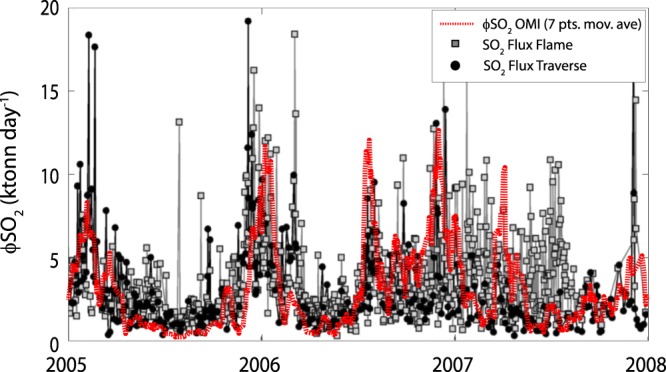
Comparison between OMI-derived SO_2_ flux (eqs 5 and 6) and ground measurements provided by Flame network and traverse method, respectively (modified from Salerno *et al.*^55^).

To convert the mass burden (M_SO2_) into SO_2_ flux (*ϕ*SO_2_) we used the “delta-M” method^65^, according which:

6 $${\varphi }_{SO2}=\frac{1}{\tau }\cdot \frac{{M}_{i}-({M}_{i-1}\cdot {e}^{-\frac{{\rm{\Delta }}t}{\tau }})}{1-{e}^{-\frac{{\rm{\Delta }}t}{\tau }}}$$

where Δt is the time interval (days) between two consecutive mass estimates M_i_ and M_i−1_, and τ is the SO_2_ e-folding time, an SO_2_ loss term that takes into account chemical, transport and dilution processes inside the plume^67^. We settled τ equal to 2 days, which has been proved to be an optimized value for a continuously degassing volcano in a stable wind regime^68^. The resulting time series consists of 754 discrete measurements of *ϕSO*_2_ (Supplementary Dataset) which are compared with ground measurements (Fig. 6) obtained with the FLAME network and traverse method for the same period^55^. The uncertainty in the estimation of *ϕSO*_2_ using eq. 6 derives from the complex combination of the uncertainties in the selection of the proper center of mass altitudes (hence M_SO2_) as well as on the assumed SO_2_ loss term (τ). Although the exact error of each of these parameters remains difficult to quantify (since it may vary from image to image) the excellent agreement with ground measurements, in terms of average values (3259 tonnes day^−1^ from OMI; 3530 tonnes day^−1^ from Flame network; 3250 tonnes day^−1^ from traverse method) and trend (Fig. 6), provides a first order validation for *ϕSO*_2_ derived from OMI whose uncertainty is considered comparable with ground measurements (i.e. ±30%^55^).

Finally, to convert the *ϕ*SO_2_ into magma supply rate (Q_in_) we used the eq. (2) by assuming a total sulfur loss ΔS, equal to 3600 ppm. This value is derived from the maximum initial S content measured on melt inclusion of Mt Etna^69^ and assuming a complete volatile loss during magma ascent. Note that the assumption of a lower initial sulphur content or an incomplete loss of the volatile during the rise of the magma, produces higher values of Q_in_. Consequently, the results from eq. 1 are to be considered minimum values on which the uncertainty of *ϕSO*_2_ (±30%) is propagated.

## Supplementary information

Supplementary Dataset 1

## Data Availability

The satellite datasets (VRP and *ϕ*SO_2_ time series) and the cumulative volumes (Vin and Vout) are available as Supplementary Datasets.

## References

[1] Francis P, Oppenheimer C, Stevenson D (1993). Endogenous growth of persistently active volcanoes. Nature.

[2] Biggs J, Pritchard ME (2017). Global volcano monitoring: What does it mean when volcanoes deform?. Elements.

[3] Devine JD, Sigurdsson H, Davis AN, Self S (1984). Estimates of sulfur and chlorine yield to the atmosphere from volcanic eruptions and potential climatic effects. J. Geophys. Res..

[4] Allard P, Carbonnelle J, Métrich N, Loyer H, Zettwoog P (1994). Sulphur output and magma degassing budget of Stromboli volcano. Nature.

[5] Harris AJL, Dehn J, Calvari S (2007). Lava effusion rate definition and measurement: A review. Bull. Volcanol..

[6] Pieri D, Baloga SM (1986). Eruption rate, area, and length relationships for some Hawaiian lava flows. J. Volcanol. Geotherm. Res..

[7] Harris AJL, Baloga SM (2009). Lava discharge rates from satellite-measured heat flux. Geophys. Res. Lett..

[8] Coppola D, Laiolo M, Piscopo D, Cigolini C (2013). Rheological control on the radiant density of active lava flows and domes. J. Volcanol. Geotherm. Res..

[9] Delaney PT, McTigue DF (1994). Volume of magma accumulation or withdrawal estimated from surface uplift or subsidence, with application to the 1960 collapse of Kilauea Volcano. Bull. Volcanol..

[10] Johnson DJ, Sigmundsson F, Delaney PT (2000). Comment on ‘‘Volume of magma accumulation or withdrawal estimated from surfaceuplift or subsidence, with application to the 1960 collapse of KilaueaVolcano’’ by Delaney, T. T. & McTigue T. F. Bull. Volcanol..

[11] Rivalta E, Segall P (2008). Magma compressibility and the missing source for some dike intrusions. Geophys. Res. Lett..

[12] Segall P (2013). Volcano deformation and eruption forecasting. Geol. Soc., Lond. Sp. Pub..

[13] M-Kilbride B, Edmonds M, Biggs J (2016). Observing eruptions of gas-rich, compressible magmas from space. Nat. Comm..

[14] Chaussard E, Amelung F, Aoki Y (2013). Characterization of open and closed volcanic systems in Indonesia and Mexico using InSAR time series. J. Geophys. Res..

[15] Swanson DA (1985). Forecasts and predictions of eruptive activity at Mount St. Helens, USA: 1975–1984. J. Geodyn..

[16] Sturkell E (2006). Volcano geodesy and magma dynamics in Iceland. J. Volcanol. Geotherm. Res..

[17] Nooner SL, Chadwick JWW (2016). Inflation-predictable behaviour and co-eruption deformation at Axial Seamount. Science.

[18] Rose, W. I., Palma, J. L., Delgado, H. & Varley, N. Open-vent volcanism and related hazards: Overview. In Rose, W. I., Palma, J. L., Delgado, H. & Varley, N., eds., Understanding Open-Vent Volcanism and Related Hazards: *Geological Society of America Special Paper* **498**, p. vii–xiii; 10.1130/2013.2498(00) (2013).

[19] Shinohara H (2008). Excess degassing from volcanoes and its role on eruptive and intrusive activity. Review of Geophysics.

[20] Wallace PJ (2001). Volcanic SO2emissions and the abundance and distribution of exsolved gas in magma bodies. J. Volc. Geotherm. Res..

[21] Dzurisin, D. A comprehensive approach to monitoring volcano deformation as a window on the eruption cycle. *Rev Geophys* **41** (1); 10.1029/2001RG000107 (2001).

[22] Dvorak JJ, Dzurisin D (1993). Variations in magma supply rate at Kilauea Volcano, Hawaii. J. Geophys. Res..

[23] Allard P (1997). Endogenous magma degassing and storage at Mount Etna. Geophys. Res. Lett..

[24] Harris AJL, Stevenson D (1997). Magma budgets and steady‐state activity of Vulcano and Stromboli. Geophys Res Lett.

[25] Oppenheimer C, McGonigle AJS, Allard P, Wooster MJ, Tsanev V (2004). Sulfur, heat, and magma budget of Erta ‘Ale lava lake, Ethiopia. Geology.

[26] Genco R, Ripepe M (2010). Inflation-deflation cycles revealed by tilt and seismic records at Stromboli volcano. Geophys. Res. Lett..

[27] Nishimura T (2009). Ground deformation caused by magma ascent in an open conduit. J. Volcanol..

[28] Patrick M, Anderson KR, Poland M, Orr TR, Swanson DA (2015). Lava lake level as a gauge of magma reservoir pressure and eruptive hazard. Geology.

[29] Neri M (2009). Deformation and eruptions at Mt Etna (Italy): A lesson from 15 years of observations. Geophys. Res. Lett..

[30] Valade, S. *et al*. Tracking dynamics of magma migration in open-conduit systems. *Bull. Volcanol*. **78** (11); 10.1007/s00445-016-1072-x (2016).

[31] Ripepe M (2015). Volcano seismicity and ground deformation unveil the gravity-driven magma discharge dynamics of a volcanic eruption. Nature Commun.

[32] Ripepe M (2017). Forecasting Effusive Dynamics and decompression rates by magmastatic model at Open-vent Volcanoes. Sci Rep.

[33] Allard P, Behncke B, D’Amico S, Neri M, Gambino S (2006). Mount Etna 1993–2005: Anatomy of an evolving eruptive cycle. Earth Sci. Rev..

[34] Harris AJL (2000). Effusion rate trends at Etna and Krafla and their implications for eruptive mechanisms. J. Volcanol. Geotherm. Res..

[35] Steffke AM, Harris AJL, Burton M, Caltabiano T, Salerno GG (2011). Coupled use of COSPEC and satellite measurements to define the volumetric balance during effusive eruptions at Mt. Etna, Italy. J Volcanol Geotherm Res.

[36] D’Aleo, R. *et al*. Understanding the SO2 Degassing Budget of Mt Etna's Paroxysms: First Clues From the December 2015 Sequence. *Front. Earth Sci*. **6** (239); 10.3389/feart.2018.00239 (2019).

[37] Pepe A, Sansosti E, Berardino P, Lanari R (2005). On the generation of ERS/ENVISAT DInSAR time-series via the SBAS technique. Remote Sens. Lett..

[38] Bruno V (2012). Ground deformations and volcanic processes as imaged by CGPS data at Mt. Etna (Italy) between 2003 and 2008. J. Geophys. Res..

[39] Palano M, Viccaro M, Zuccarello F, Gresta S (2017). Magma transport and storage at Mt. Etna (Italy): A review of geodeticand petrological data for the 2002–03, 2004 and 2006 eruptions. J. Volcanol. Geotherm. Res..

[40] Aloisi, M., Bonaccorso, A., Cannavò, F. & Currenti, G.M. Coupled Short- and Medium-Term Geophysical Signals at Etna Volcano: Using Deformation and Strain to Infer Magmatic Processes From 2009 to 2017. *Front. Earth Sci*. **6**(109); 10.3389/feart.2018.00109. (2018).

[41] Neri M, Acocella V (2006). The 2004–2005 Etna eruption: Implications for flank deformation and structural behaviour of the volcano. J. Volcanol. Geotherm. Res..

[42] Harris AJL, Steffke A, Calvari S, Spampinato L (2011). Thirty years of satellite-derived lava discharge rates at Etna: implications for steady volumetric output. J Geophys Res.

[43] Corsaro RA, Miraglia L (2005). Dynamics of 2004–2005 Mt. Etna effusive eruption as inferred from petrologic monitoring. Geophys. Res. Lett..

[44] Burton M (2005). Etna 2004–2005: An archetype for geodynamically-controlled effusive eruptions. Geophys. Res. Lett..

[45] Falsaperla S, Barberi G, Cocina O (2014). The failed eruption of Mt. Etna in December 2005: Evidence from volcanic tremor analyses. Geochem. Geophys. Geosys..

[46] Behncke B, Calvari S, Gianmanco S, Neri M, Pinkerton H (2008). Pyroclastic density currents resulting from the interaction of basaltic magma with hydrothermally altered rock: an example from the 2006 summit eruptions of Mount Etna, Italy. Bull. Volcanol..

[47] Neri M (2006). Continuous soil radon monitoring during the July 2006 Etna eruption. Geophys. Res. Lett..

[48] Acocella, V. *et al*. Why Does a Mature Volcano Need New Vents? The Case of the New Southeast Crater at Etna. *Front. Earth Sci*. **4**(67); 10.3389/feart.2016.00067 (2016).

[49] Andronico D, Lo Castro MD, Spina L (2013). The 2010 ash emissions at the summit craters of Mt Etna: Relationship with seismo-acoustic signals. J. Geophys. Res..

[50] Aiuppa A (2010). Patterns in the recent 2007–2008 activity of Mount Etna volcano investigated by integrated geophysical and geochemical observations. Geochem. Geophys. Geosyst..

[51] Bonaccorso A (2010). Multidisciplinary investigation on a lava fountain preceding a flank eruption: The 10 May 2008 Etna case. Geochem.Geophys. Geosyst..

[52] Bonforte A, Guglielmino F, Puglisi G (2013). Interaction between magma intrusion and flank dynamics at Mt. Etna in 2008, imaged by integrated dense GPS and DInSAR data. Geochem. Geophys. Geosyst..

[53] Behncke B (2016). Lidar surveys reveal eruptivevolumes and rates at Etna, 2007–2010. Geophys. Res. Lett..

[54] Coppola D (2013). Radiative heat power at Stromboli volcano during 2000–2011: Twelve years of MODIS observations. J. Volcanol. Geotherm. Res..

[55] Salerno GC (2009). Three-years of SO2 flux measurements of Mt. Etna using an automated UV scanner array: Comparison with conventional traverses and uncertainties in flux retrieval. J. Volcanol. Geotherm. Res..

[56] Aiuppa A (2018). Tracking formation of a lava lake from ground and space: Masaya volcano (Nicaragua), 2014–2017. Geochem. Geophys. Geosyst..

[57] Coppola D., Laiolo M., Cigolini C., Donne D. Delle, Ripepe M. (2015). Enhanced volcanic hot-spot detection using MODIS IR data: results from the MIROVA system. Geological Society, London, Special Publications.

[58] Henley RW, Hughes G (2016). SO2 flux and the thermal power of volcanic eruptions. J. Volcanol. Geotherm. Res..

[59] Reath K (2019). Thermal, deformation, and degassing remote sensing time-series (A.D. 2000-2017) at the 47 most active volcanoes in Latin America: Implications for volcanic systems. J. Geophys. Res.

[60] Wooster MJ, Zhukov B, Oertel D (2003). Fire radiative energy for quantitative study of biomass burning: derivation from the BIRD experimental satellite and comparison to MODIS fire products. Remote Sens Environ.

[61] Coppola, D. *et al*. Monitoring the time-averaged discharge rates, volumes and emplacement style of large lava flows by using MIROVA system: the case of the 2014–2015 eruption at Holuhraun (Iceland). *Annals of Geophysics* **61**; 10.4401/ag-7749 (2019).

[62] Corsaro RA, Pompilio M (2004). Buoyancy‐controlled eruption of magmas at Mt Etna. Terra Nova.

[63] Krotkov, N.A., Li, C. & Leonard, P. OMI/Aura Sulphur Dioxide (SO2) Total Column Daily L2 Global Gridded 0.125 degree × 0.125 degree V3. *Greenbelt, MD, USA, Goddard Earth Sciences Data and Information Services Center* (**GES DISC**): Accessed [01032018-10032018], 10.5067/Aura/OMI/DATA2023 (2014).

[64] Laiolo, M., Massimetti, F., Cigolini, C., Ripepe, M., & Coppola, D. Long-term eruptive trends from space-based thermal and SO2 emissions: A comparative analysis of stromboli, batu tara and tinakula volcanoes. *Bull. Volcanol*. **80**(9), 10.1007/s00445-018-1242-0 (2018).

[65] Krueger AJ (1995). Volcanic sulfur dioxide measurements from the total ozone mapping spectrometer instruments. J. Geophys. Res..

[66] Carn SA, Fioletov VE, McLinden CA, Li C, Krotkov NA (2017). A decade of global volcanic SO2 emissions measured from space. Sci. Rep..

[67] Theys N (2013). Volcanic SO2 fluxes derived from satellite data: a survey using OMI, GOME-2, IASI and MODIS. Atmos. Chem. Phys..

[68] Beirle S (2014). Estimating the volcanic emission rate and atmospheric lifetime of SO2 from space: a case study for Kılauea volcano, Hawai‘i. Atmos. Chem. Phys..

[69] Clochiatti RA, Weisz J, Mosba M, Tangy JC (1992). Coexistence de verres alcalins et tholéiitiques saturés en CO2 dans les olivines des hyaloclastites d’Aci Castello (Etna, Sicile, Italie). Arguments en faveur d’un manteau anormal et d’un réservoir profound. Acta Vulcanol..

[70] Tarquini Simone, Vinci Stefano, Favalli Massimiliano, Doumaz Fawzi, Fornaciai Alessandro, Nannipieri Luca (2012). Release of a 10-m-resolution DEM for the Italian territory: Comparison with global-coverage DEMs and anaglyph-mode exploration via the web. Computers & Geosciences.

